# Sigma-1 Receptor Signaling: In Search of New Therapeutic Alternatives for Cardiovascular and Renal Diseases

**DOI:** 10.3390/ijms24031997

**Published:** 2023-01-19

**Authors:** Francisco Javier Munguia-Galaviz, Alejandra Guillermina Miranda-Diaz, Miguel Alejandro Cardenas-Sosa, Raquel Echavarria

**Affiliations:** 1 Departamento de Fisiologia, Centro Universitario de Ciencias de la Salud, Universidad de Guadalajara, Guadalajara 44340, Jalisco, Mexico; 2 Division de Ciencias de la Salud, Centro Universitario del Sur, Universidad de Guadalajara, Ciudad Guzman 49000, Jalisco, Mexico; 3 CONACYT-Centro de Investigacion Biomedica de Occidente, Instituto Mexicano del Seguro Social, Guadalajara 44340, Jalisco, Mexico

**Keywords:** sigma 1, ER signaling, CVD, CKD, AKI, ER stress, UPR, drug repurposing, chaperone

## Abstract

Cardiovascular and renal diseases are among the leading causes of death worldwide, and regardless of current efforts, there is a demanding need for therapeutic alternatives to reduce their progression to advanced stages. The stress caused by diseases leads to the activation of protective mechanisms in the cell, including chaperone proteins. The Sigma-1 receptor (Sig-1R) is a ligand-operated chaperone protein that modulates signal transduction during cellular stress processes. Sig-1R interacts with various ligands and proteins to elicit distinct cellular responses, thus, making it a potential target for pharmacological modulation. Furthermore, Sig-1R ligands activate signaling pathways that promote cardioprotection, ameliorate ischemic injury, and drive myofibroblast activation and fibrosis. The role of Sig-1R in diseases has also made it a point of interest in developing clinical trials for pain, neurodegeneration, ischemic stroke, depression in patients with heart failure, and COVID-19. Sig-1R ligands in preclinical models have significantly beneficial effects associated with improved cardiac function, ventricular remodeling, hypertrophy reduction, and, in the kidney, reduced ischemic damage. These basic discoveries could inform clinical trials for heart failure (HF), myocardial hypertrophy, acute kidney injury (AKI), and chronic kidney disease (CKD). Here, we review Sig-1R signaling pathways and the evidence of Sig-1R modulation in preclinical cardiac and renal injury models to support the potential therapeutic use of Sig-1R agonists and antagonists in these diseases.

## 1. Introduction

The stress caused by diseases changes the molecular behavior of the cells in different organ systems, activating protective mechanisms that promote survival over death. These protection processes rely on signaling pathways acting synchronously via enzymatic, ionic, and protein effectors such as chaperone proteins [1]. Martin et al. initially identified the Sigma 1 receptor (Sig-1R) as a mediator of the physiological and behavioral effects of opiate benzomorphans like N-allylnormetazocine [2]. However, the Sig-1R is not an opioid receptor but an intracellular ligand-operated chaperone that regulates the activity of other proteins, and its mechanisms of action are particularly relevant under stress conditions as Sig-1R operates to normalize physiological responses [3,4,5,6]. 

The function of Sig-1R has been studied in Sig-1R knock-out (KO) mice models, observing the development of multiple pathological phenotypes [7,8,9,10,11,12]. Sig-1R is involved in cognitive, behavioral, and synaptic functions, neurogenesis, pain, and addiction [13]. Moreover, Sig-1R ligands have been postulated as protective agents to treat specific psychiatric and neurodegenerative disorders [14,15]. Despite the Central Nervous System (CNS) being the target site with the most significant progress regarding Sig-1R therapeutics, cellular and preclinical models provide evidence of the potential for targeting Sig-1R signaling in other organs, including the heart and the kidney. Sig-1R KO mice exhibit significant alterations in mitochondrial dynamics and cardiac dysfunction [12]. In addition, Sig-1R activation is cardioprotective in preclinical models of cardiac injury such as myocardial ischemia-reperfusion injury (IRI), heart failure (HF), hypertrophy, cardiac fibrosis, cardiomyopathy, and cardiovascular complications associated with Severe Acute Respiratory Syndrome Coronavirus 2 (SARS-CoV-2) infection [16,17,18,19,20,21,22,23,24,25,26,27,28,29]. A systematic review by Lewis et al. reinforced the potential of Sig-1R targeting to treat cardiovascular diseases (CVDs), notably cardiac hypertrophy [30]. In the kidney, Sig-1R agonism ameliorates IRI and fibrosis, essential mechanisms in the pathophysiology of acute kidney injury (AKI), chronic kidney disease (CKD), and transplantation, and it plays a role in modulating sex-dependent renoprotection [31,32,33].

CVDs and renal diseases are among the leading causes of death worldwide [34,35]. Moreover, there is a close link between cardiovascular and kidney pathologies, as the disease of one organ often causes dysfunction of the other and leads to both failures [36]. Despite current efforts, there is an urgent need for novel therapeutic alternatives to reduce their progression to advanced stages. The Sig-1R is an interorganelle signaling regulator that can interact with various ligands and proteins to elicit diverse cellular responses, thus, making it a potential target for pharmacological modulation in pathologies affecting the heart and the kidneys [37]. Here, we review Sig-1R signaling pathways and the evidence of Sig-1R modulation in preclinical cardiac and renal injury models to suggest a promising therapeutic use of Sig-1R agonists and antagonists in these diseases. 

## 2. Sig-1R Structure and Intracellular Localization

There are two subtypes of sigma receptors: Sig-1R and sigma-2 (Sig-2R). Sig-1R, successfully cloned in 1996 from guinea pig liver, is a 25 kDa ligand-operated signaling protein, structurally unrelated to other known mammalian proteins, that shares homology with a fungal protein involved in sterol synthesis but lacks its activity [38].

**Figure 1:**
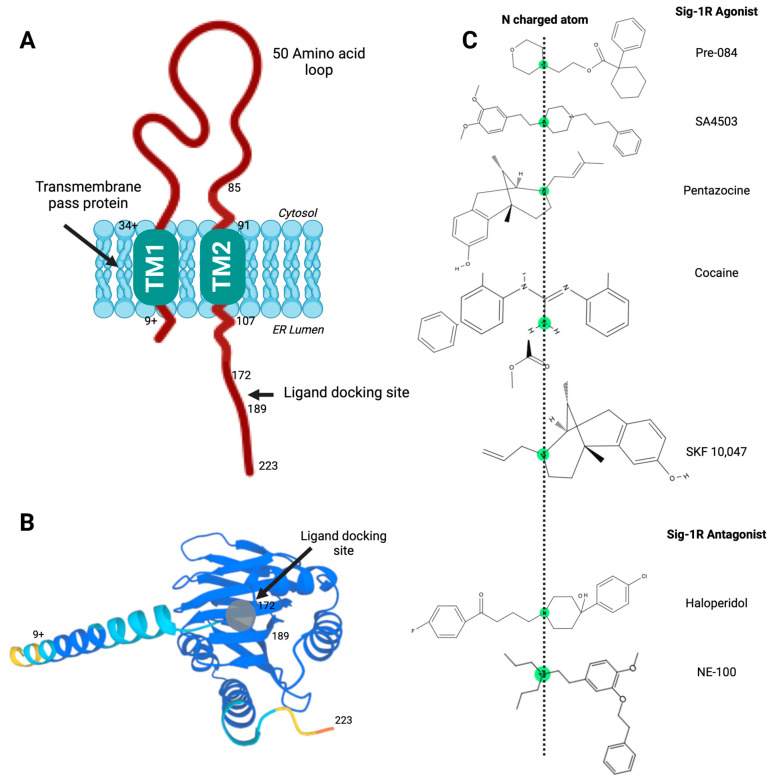
Sig-1R structure and ligand-receptor interactions. (**A**) Proposed model of Sig-1R with two transmembrane helices and a C-terminal ligand docking site; (**B**) Sig-1R tertiary structure with a ligand docking site predicted to accommodate agonist and antagonist ligands; (**C**) High-affinity ligands of Sig-1R contain a nitrogen atom connected to long alkyl chains essential for the molecular coupling of such diverse compounds and their stereoisomeric forms. Pharmacophore model for the Sig-1R ligands (agonists and antagonists), in which this central basic amine nitrogen atom, surrounded by two hydrophobic features, interacts with the bulky alternate chains of ligands without altering their binding affinity.

The *SIGMAR1* gene maps to human chromosome 9p13.3, and its alternative splicing results in transcript variants encoding distinct isoforms. The Sig-1R isoform chosen as the canonical sequence has 223 amino acids with an N-terminal domain containing an endoplasmic reticulum (ER) retention signal and a C-terminal hydrophobic domain similar to regions that bind steroids [39]. In addition, hydrophobicity profiles and experimental data suggest that Sig-1R has two transmembrane helices (Figure 1A) [40,41,42]. However, ligand-bound Sig-1R crystal structures exhibit a trimeric architecture with an N-terminus in the ER lumen, followed by a single transmembrane domain and the C-terminus on the cytosolic side of the ER membrane [43]. Moreover, evidence from ultrastructural studies further supports the existence of a transmembrane domain between amino acids 1–80, but with the N-terminus on the cytosolic side and the C-terminus facing the ER lumen [44]. Hence, the Sig-1R insertion topography remains inconclusive.

The importance of the C-terminal domain for ligand binding became apparent when a Sig-1R splice variant that lacks thirty-one amino acids encoded by exon 3 was nonfunctional in ligand binding assays [45]. Soon after, Seth et al. used chemical modification and site-directed mutagenesis to identify the anionic amino acid residues D126 and E172 in the Sig-1R C-terminal domain as critical for ligand binding [46]. Furthermore, the proximity of hydrophobic steroid binding domain-like (SBDL) regions, sharing significant homology with fungal sterol isomerase, form Sig-1R ligand-binding areas. Specifically, the regions comprising amino acids 91-109 (SBDLI) and 176–194 (SBDLII) participate in Sig-1R ligand binding [47]. Hence, the Sig-1R C-terminal domain contains a ligand docking site (Figure 1A,B) and mediates Sig-1R oligomerization [43].

The exact intracellular location and dynamics of Sig-1R are still under study. Sig-1R mainly resides in the ER, particularly in the mitochondria-associated ER membrane domain (MAM), but there are reports of its presence in the plasma membrane, the mitochondria, and the nucleus [7,48,49,50]. Furthermore, multiple studies have shown that Sig-1R subcellular location depends on the cell type and changes upon activation, translocating from the MAM to the plasma membrane, where it can communicate with other signaling proteins or the nucleus to modulate gene transcription [48,49,51,52,53]. Nonetheless, Sharma et al. recently used protease protection assays and a glycosylation mapping approach to demonstrate that Sig-1R is confined to the ER, with minimal displacement to the plasma membrane even in the presence of Sig-1R agonists or ER stress [54]. 

## 3. Sig-1R Ligands

Sig-1R binds structurally diverse ligands such as benzomorphans, neuroleptics, antidepressants, psychoactive drugs, and steroids (Table 1). Steroids are the predominant endogenous modulators of Sig-1R, particularly those synthesized in the nervous system, like pregnenolone-sulfate (PREG-S) and dehydroepiandrosterone sulfate (DHEA-S) [3,55]. Progesterone, testosterone, cholesterol, and endogenous long alkyl chain primary amines of the sphingolipid family, such as D-erythro-sphingosine and sphinganine, also exhibit affinity towards Sig-1Rs [55,56,57,58].

Sig-1R ligands can be agonists or antagonists, and, as such, they can elicit different cellular responses by allowing opposing ligand-mediated Sig-1R conformational changes and the induction of distinct oligomeric states in the receptor [59,60,61]. Sig-1R endogenous agonists like PREG-S and DHEA-S enhance physiological responses or normalize alterations produced under pathological conditions, similarly to other synthetic agonists such as PRE-084, SA4503, Pentazocine, 1,3-di-o-tolyl-guanidine (DTG), and N-allylnormetazocine (SKF-10047). On the other hand, antagonists like progesterone and psychoactive substances such as cocaine, haloperidol, fluvoxamine, or sertraline counteract these effects [62,63,64,65,66,67].

Presently, the molecular mechanisms mediating the varied Sig-1R ligand-receptor interactions remain a subject of investigation. High-affinity ligands of Sig-1R contain a nitrogen atom connected to long alkyl chains essential for the molecular coupling of such diverse compounds and their stereoisomeric forms (Figure 1C). Glennon RA et al. proposed a first 2D-pharmacophore model pharmacophore model for the Sig-1R ligands in which this central basic amine nitrogen atom, surrounded by two hydrophobic features, interacts with the bulky alternate chains of ligands without altering their binding affinity [68]. Although this initial qualitative model has helped design Sig-1R ligands, the availability of Sig-1R’s crystal structure prompted the advancement of other models to develop and validate new molecules as Sig-1R agonists and antagonists [61,69].

## 4. Receptor Oligomerization and the Sig-1R-BiP Complex

Sig-1R exists in fluctuating oligomeric states mediated by the action of its ligands [60,61,70]. Sig-1R binding of antagonists favors the higher-order receptor oligomers, while the agonists enhance the formation of small oligomers in their monomeric and dimeric states. The C-terminal domain mediates Sig-1R oligomerization [43], a GXXXG motif essential for stabilizing the functional oligomeric states and ligand binding [70].

**Figure 2:**
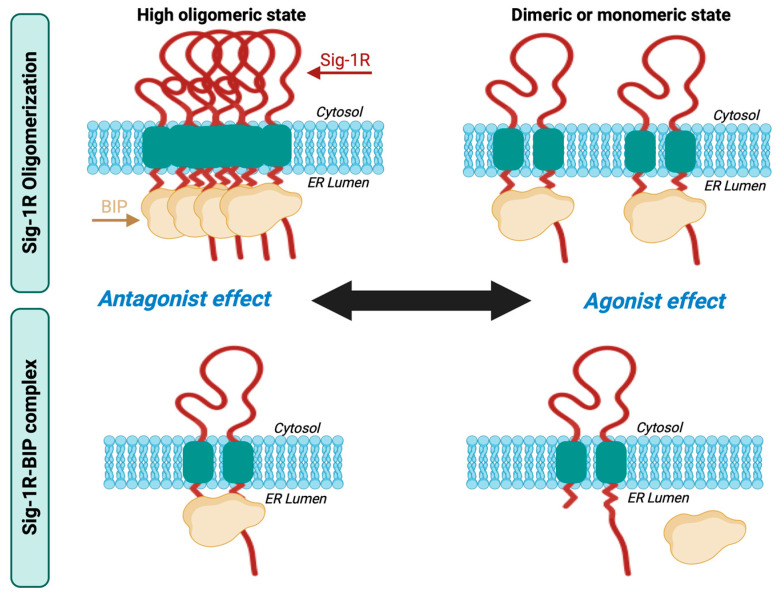
Sig-1R oligomerization and Sig-1R-BiP complex formation. Sig-1R ligands fine-tune their fluctuating oligomeric states in which antagonists promote the stabilization of the Sig-1R-BiP complex, and agonists favor Sig-1R dissociation from BiP.

Ligand-dependent modulation of Sig-1R multimerization promotes the formation of a complex between Sig-1R and BiP, another ER molecular chaperone, an interaction decisive for its activity, interaction with other proteins, and activation of downstream signaling pathways [41]. BiP is an ATP-dependent chaperone essential for restoring homeostasis after imbalances in protein folding and degradation result in the accumulation of unfolded proteins in the ER [71]. Sig-1R is not a BiP substrate; instead, Sig-1R possesses innate chaperone activity, and the interaction between its C-terminal domain and BiP maintains it in its resting state [41,72]. Sig-1R antagonists like haloperidol promote oligomerization and inhibit Sig-1R-BiP dissociation, whereas agonists such as pentazocine destabilize Sig-1R oligomers upon binding, allowing Sig-1R to separate from BiP and initiate its signaling activities (Figure 2) [6,41].

## 5. Sig-1R Signaling Pathways

### 5.1. Cytoplasmic Organelles

#### 5.1.1. ER-Mitochondria Interface

**Figure 3:**
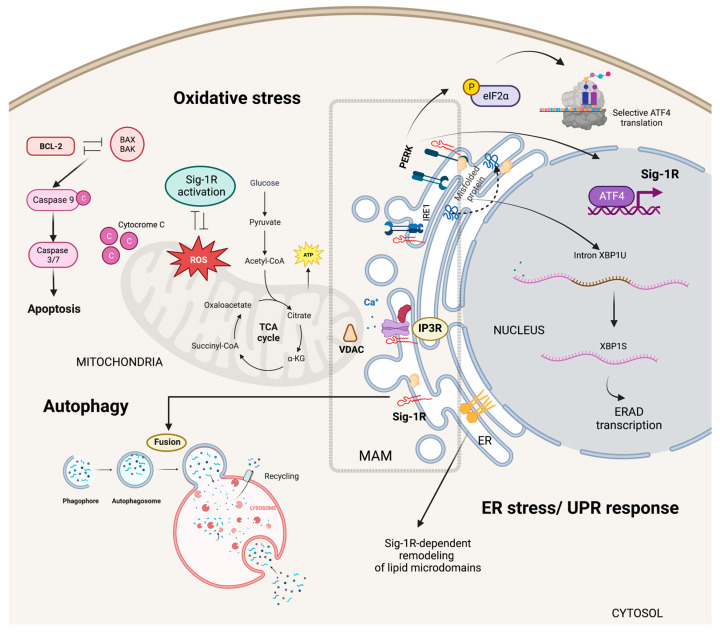
Sig-1R signaling in cytoplasmic organelles. Sig-1R is enriched at ER MAMs where microdomains at focal contacts between ER and mitochondria allow calcium release from the ER via IP3Rs into the mitochondria regulating calcium signaling between these two organelles. Sig-1R also plays a dual role in regulating oxidative stress responses and acts as an ER lipid scaffolding protein able to interact with and remodel cholesterol-rich microdomains. In response to ER stress, Sig-1R increases the calcium leak from the ER into the mitochondria to strengthen mitochondrial bioenergetics and enters a positive transcriptional loop that increases Sig-1R expression through the PERK/eIF2-α/ATF4 pathway. Sig-1R modulates the autophagosome-lysosome fusion step without altering PINK1/Parkin signaling or autophagosome biogenesis.

Sig-1R is an ER-localized type II membrane protein enriched at MAMs where the presence of microdomains at focal contacts between ER and mitochondria allows calcium release from the ER via inositol 1,4,5-trisphosphate receptors (IP3Rs) into the mitochondria coordinating the regulation of calcium signaling between these two organelles (Figure 3) [41,54]. Calcium influx increases ATP production by activating tricarboxylic acid cycle enzymes, regulating mitochondrial bioenergetics, free radical formation, and stress survival. The relevance of IP3R-mediated calcium uptake for Sig-1R signaling activation is further supported by in vitro experiments in which the expression of a truncated form of Sig-1R unable to interact with IP3Rs suppresses mitochondrial ATP production and elicits apoptosis [73]. Moreover, overexpression of another Sig-1R variant, E102Q, that promotes its detachment from ER membranes results in cytoplasmic aggregation, reduced ATP production, and cell death [74].

Sig-1R plays a dual role in regulating oxidative stress responses as evidence of increased reactive oxygen species (ROS) production after Sig-1R overexpression contrasts with reports suggesting Sig-1R activation ameliorates oxidative stress [75,76,77,78,79]. In addition, organs and cells of Sig-1R KO mice have higher ROS levels than wild-type mice, and Sig-1R upregulates the transcription of antioxidant enzymes, further supporting the antioxidant role of Sig-1R signaling [60]. The nuclear factor (erythroid-derived 2)-like 2 (NRF2) and zinc finger protein 179 (ZNF179) are some of the Sig-1R downstream effectors protecting mitochondria from oxidative stress [80,81,82].

Mitochondrial metabolism and oxidative stress are closely linked to cell survival and apoptosis [83]. Sig-1R signaling protects against oxygen-glucose deprivation and hydrogen peroxide-induced apoptosis through the upregulation of the apoptosis regulator BCL2 and, at least in part, via a ROS/nuclear factor kappa b (NFκB) mechanism [84,85]. Similarly, Sig-1R offered protection against glutamate-induced cell apoptosis by regulating intracellular calcium, BAX levels, and caspase-3 activation [86]. Conversely, Sig-1R ligands’ pro-apoptotic behavior associates with caspase activation, calcium-dependent activation of phospholipase C (PLC), mitogen-activated protein kinases (MAPKs), phosphoinositide-3-kinase (PI3K)/AKT signaling, p53 and the hypoxia-inducible factor 1 alpha (HIF-1α) pathways [87,88,89].

#### 5.1.2. Specialized ER Lipid Microdomains

ER synthesis of phosphatidylserine, steroids, and sphingolipids depends on MAMs and their unique levels of ceramides and cholesterol compared to other membranes [90,91]. Sig-1R can bind to sterols and sphingolipids, and cholesterol induces its clustering in lipid bilayers, acting as an ER lipid scaffolding protein able to interact with and remodel cholesterol-rich microdomains (Figure 3) [57,58,92,93]. Consequently, Sig-1R can selectively recruit multiple effector proteins to these microdomains and, thus, modulate their activity [15,94,95].

#### 5.1.3. ER Stress Responses

ER stress results from unfolded protein accumulation or ER calcium depletion and leads to activation of the unfolded protein response (UPR) [96]. Inositol requiring enzyme 1 (IRE1), activating transcription factor 6 (ATF6), and protein kinase RNA-like endoplasmic reticulum kinase (PERK) are ER-resident proteins that control the UPR through the activation of transcriptional and translational mechanisms that reduce protein synthesis while increasing protein-folding efficiency and misfolded protein degradation.

Metabolic changes that critically depend on calcium influx from the ER into mitochondria and the upregulation of ER chaperones mark the early phases of ER stress [97,98,99]. Sig-1R increases the calcium leak from the ER into the mitochondria and enhances mitochondrial bioenergetics shortly after ER stress induction with tunicamycin [100]. Moreover, pentazocine upregulates PERK, activating transcription factor 4 (ATF4), ATF6, IRE1, and the C/EBP Homologous Protein (CHOP) expression in experimental models of diabetic retinopathy [101]. Transcriptomic analysis of isolated retinal Müller glial cells from Sig-1R KO mice revealed significant changes in the expression of ER stress genes, particularly ATF6, highlighting Sig-1R’s role in ER stress regulation [102].

ER stress induces Sig-1R expression through the PERK/Eukaryotic translation initiation factor 2A (eIF2-α)/ATF4 pathway [103]. In addition, PERK/eIF2-α/ATF4 pathway activation inhibits caspase-4 processing and mitigates cell death signaling. ATF4 is a transcription factor able to interact with the *SIGMAR1* gene in manners that are PERK-dependent, in response to ER stress, or PERK-independent, upon fluvoxamine binding to Sig-1R [104]. Mitochondrial ROS are the primary activators of the ER stress sensor IRE1 at the MAM [105]. Under stress conditions, Sig-1R also stabilizes IRE1, thus, extending the activation of the IRE1-X-box binding protein 1 (XBP1) signaling pathway and XBP1 mRNA splicing to enhance cell survival (Figure 3) [106].

#### 5.1.4. Autophagy and Mitophagy

Proteins, lipids, and dysfunctional organelles are degraded and recycled through lysosome-mediated autophagy. ER MAMs are essential for the early phases of autophagy since treatments that blunt the ER-mitochondria communication prevent autophagosome nucleation [107,108]. Interestingly, Sig-1R downregulation or cell treatment with a Sig-1R antagonist results in autophagosome accumulation and enhanced ER stress [93,109,110,111,112].

During mitophagy, the clearance of mitochondria, the serine/threonine kinase PTEN-induced kinase 1 (PINK1) collaborates with Parkin, an E3 ubiquitin ligase, to target damaged mitochondria to the lysosome for degradation [113]. Sig-1R KO cells have impaired mitophagy and increased autophagosome markers LC3-II and sequestosome 1 (SQSTM1). Sig-1R modulates the autophagosome-lysosome fusion step without altering PINK1/Parkin signaling or autophagosome biogenesis (Figure 3) [114].

### 5.2. Nucleus

**Figure 4:**
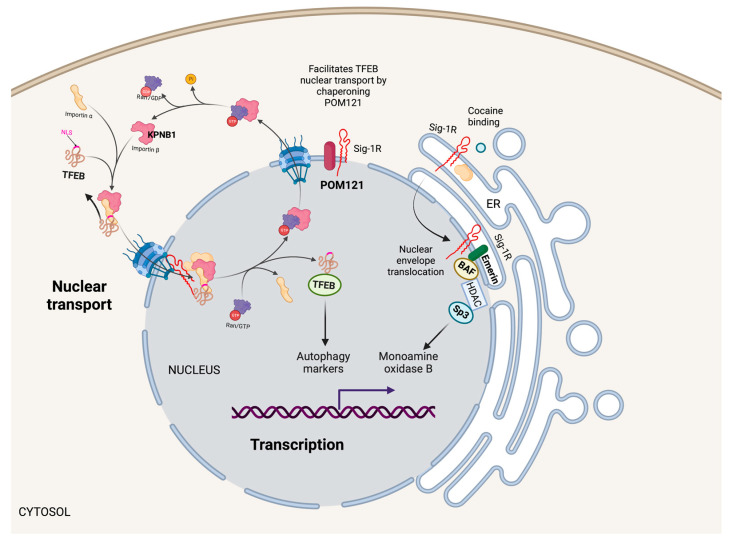
Nuclear Sig-1R signaling pathways. Sig-1R localizes in the nuclear envelope and inside the nucleus to regulate nuclear functions. For example, Sig-1R increases monoamine oxidase B transcription by forming a Sig-1R/Emerin/BAF/HDAC/SP3 complex that binds to its promoter. Additionally, Sig-1R facilitates the nuclear transport of TFEB through the nuclear pore by chaperoning POM121, a nucleoporin that recruits KPNB1, influencing autophagy.

Although Sig-1R is a membrane protein predominantly residing in the ER, its presence in the nuclear envelope and inside the nucleus suggests that Sig-1R molecular functions extend to nuclear regulation (Figure 4) [115,116]. Yasuo et al. evidenced that leptomycin B, an exportin 1 inhibitor, and thapsigargin, an ER-stress inducer, retain Sig-1R in the nucleus in collaboration with p62 [117]. Moreover, the in vitro findings correlate with observations of Sig-1R consistently co-localizing with neuronal nuclear inclusions in the brains of patients with neurodegenerative diseases and intranuclear Marinesco bodies in normal, aged brains.

#### 5.2.1. Transcription

Cocaine, a Sig-1R agonist, triggers Sig-1R shuttling to the nuclear envelope, where it binds to Emerin and recruits chromatin-remodeling molecules like laminin A/C, barrier-to-autointegration factor (BAF), and histone deacetylase (HDAC), to form a complex with the gene repressor specific protein 3 (SP3) [53]. Moreover, cocaine increases the association of Sig-1R/Emerin/BAF/HDAC/SP3 with the monoamine oxidase B promoter, thus, explaining why cocaine suppresses monoamine oxidase B expression in the brain of wild-type mice but not Sig-1R KOs (Figure 4).

#### 5.2.2. Nuclear Transport

The cytoplasmic accumulation of Ran-activating protein (RanGAP) caused by the binding of the (G4C2)-RNA repeat expansion from C9orf72 chromosome to RanGAP at the nuclear pore is characteristic in some patients with amyotrophic lateral sclerosis and frontotemporal dementia [118]. Evidence from cellular models and Drosophila supports a role for Sig-1R in regulating nuclear-cytoplasmic transport since Sig-1R directly binds (G4C2)-RNA repeats and reverses aberrant cytoplasmic accumulation of Ran by interacting with RanGAP and stabilizing nuclear pore proteins [119]. Similarly, the (G4C2)-RNA repeat expansion disrupts the nucleocytoplasmic transport of transcription factor EB (TFEB), an essential transcription factor for autophagy. Sig-1R facilitates TFEB transport through the nuclear pore by chaperoning nuclear envelope pore membrane protein POM 121 (POM121), a nucleoporin that recruits importin subunit beta 1 (KPNB1) (Figure 4). Sig-1R overexpression or treatment with the Sig-1R agonist pridopidine increased TFEB nuclear levels, KPNB1, and autophagy markers [120]. Evidence supports a protective role of Sig-1R in G4C2 repeat expansion disorders with impaired protein clearance and suboptimal autophagy by regulating nuclear transport and reducing toxic protein accumulation leading to cell death.

### 5.3. Plasma Membrane

**Figure 5:**
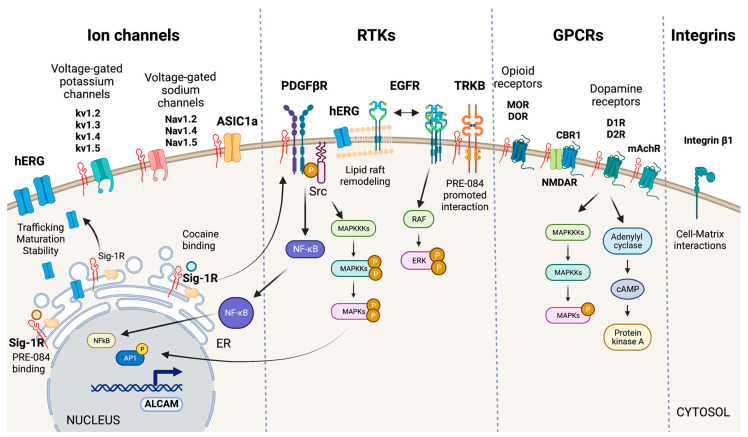
Sig-1R interactions with plasma membrane signaling proteins. Sig-1R interacts with ion channels (hERG, voltage-gated potassium and sodium channels, and ASIC1a), RTKs (PDGF-βR, EGFR, TRKB), GPCRs (MOR, DOR, D1R, D2R, CBR1, and mAchR), and integrins (integrin β1) at the plasma membrane to regulate their activities and elicit intracellular signaling cascades.

Multiple studies report that Sig-1R interacts with ion channels, receptor tyrosine kinases (RTKs), G protein-coupled receptors (GPCRs), and integrins at the plasma membrane to regulate their activities (Figure 5) [121]. However, whether Sig-1R shuttles to the plasma membrane to perform these physical interactions or ER-located Sig-1R interacts with these proteins via Sig-1R’s proximity to the plasma membrane remains to be established and has proved to be technically challenging [54].

#### 5.3.1. Ion Channels

The effects of Sig-1R ligands on potassium, calcium, sodium, and chloride currents initially proposed a functional relationship between Sig-1R and ion channels from diverse molecular classifications, and there is experimental evidence of Sig-1R interaction with voltage-gated potassium and sodium channels and acid-sensing ion channels (ASICs) (Figure 5) [37,40,122,123,124].

hERG encodes a voltage-dependent potassium channel essential for normal electrical activity in the heart as it regulates cardiac repolarization [125]. Sig-1R ligands reversibly inhibit hERG. In contrast, Sig-1R expression increases hERG current density through the post-translational regulation of channel subunit maturation and stability [126]. Crottes et al. suggest that Sig-1R controls hERG trafficking and increases the number of functional channels at the plasma membrane. Moreover, Sig-1R might influence hERG current through changes in hERG’s localization within the plasma membrane since there is evidence that hERG translocation into lipid rafts affects potassium channel gating [127].

Additionally, Sig-1R interacts with the integral membrane voltage-gated potassium channels Kv1.2, Kv1.3, Kv1.4, and Kv1.5 [40,62,128]. In motor neurons, Sig-1R colocalizes with Kv1.2, and Sig-1R-Kv1.2 interactions determine neuronal and behavioral responses to cocaine [7,62]. Sig-1R also changes Kv1.3 voltage-gated potassium channels kinetics’ but not the sensitivity to receptor ligands which contrasts with Sig-1R’s distinct and ligand-dependent functional interactions with the Kv1.4 and Kv1.5 channels [40,128].

Sig-1R modulates various voltage-gated sodium channels like Nav1.2, Nav1.4, and Nav1.5 [129,130,131]. Johannessen M et al. studied Sig-1R modulation of the heart voltage-gated sodium channel Nav1.5 associated with cardiac excitability [131]. They found that Sig-1R agonists and antagonists reversibly inhibit Nav1.5 channels in variable grades, but none alters channel kinetics. Meanwhile, progesterone inhibits Sig-1R-mediated modulation of Nav1.5a induced by DTG, PB28, SKF-10047, and dimethyltryptamine. This voltage-gated sodium channel regulation mechanism by progesterone seems relevant to changes in cardiovascular function during endocrine transitions [132].

Sig-1R directly interacts with ASICs, proton-gated cation channels activated by acidic pH conditions, to modulate their function [123]. The ASIC1a subtype, activated by ischemia-induced acidosis, has high calcium permeability [133]. Sig-1R stimulation with its ligands inhibits ASIC1a–induced calcium influx in rat cortical neurons [134]. HF induces changes in ASICs in sensory neurons innervating skeletal muscle, and therapeutic inhibition of ASIC1a recovers heart function after IRI [135,136]. Unfortunately, the potential role of Sig-1R modulation of ASICs in ischemic processes that affect the heart and the kidneys remains largely unexplored.

#### 5.3.2. RTK Signaling

RTKs are cell-surface transmembrane proteins and signal transducers that regulate essential processes by phosphorylating tyrosine residues on their intracellular substrates [137]. Sig-1R interacts with the RTKs tropomyosin receptor kinase B (TRKB), epidermal growth factor receptor (EGFR), and platelet-derived growth factor beta receptor (PDGF-βR) (Figure 5).

TRKB is a receptor for brain-derived neurotrophic factor (BDNF) important for synaptic transmission, neurogenesis, learning, and cognition. Sig-1R agonists upregulate BNDF to exert protective effects [138,139]. Moreover, the Sig-1R agonist PRE-084 enhances its interaction with TRKB and promotes neurite elongation in cerebellar granule neurons [140]. The cardiovascular system expresses the BDNF/TRKB pathway, and this signaling cascade participates in the development of CVDs, including coronary artery disease, HF, cardiomyopathy, and hypertension [141]. Moreover, BDNF exerts cardioprotection after myocardial infarction through a CNS-mediated pathway [142].

Evidence points to a role for Sig-1R in remodeling lipid rafts to interact with transmembrane receptors and other signaling molecules in the plasma membrane [92,121,143]. EGFR activation is critical in normal cardiac development, blood pressure regulation, endothelial dysfunction, atherogenesis, cardiac remodeling, AKI, and CKD [144,145,146,147]. Non-raft regions are richer in EGFRs than lipid rafts in PC12 cells overexpressing Sig-1R and reducing EGFR localization to lipid rafts through increased Sig-1R or by disrupting cholesterol-containing rafts using methyl-beta-cyclodextrin results in heightened downstream signaling, extracellular signal-regulated kinase (ERK) phosphorylation, and neuritogenesis [148]. Furthermore, Sig-1R alters lipid rafts composition of cholesterol and gangliosides GM1, GM2, and GD1a. Similarly, cocaine promotes the shuttling of Sig-1R from the ER MAM to the plasma membrane, where it directly binds PDGF-βR to induce its phosphorylation via SRC-kinase activation. Sig-1R-dependent signaling downstream of PDGF-βR leads to NFκB and MAPKs activation, increased transcription of activated leukocyte cell adhesion molecule (ALCAM), and leukocyte transmigration across the blood-brain barrier [149].

#### 5.3.3. GPCR Signaling

Sig-1R undoubtedly modulates GPCRs as evidenced by its effects on mu-opioid receptors (MORs), delta-opioid receptors (DORs), muscarinic acetylcholine receptors (mAchRs), cannabinoid receptor 1 (CBR1), and dopamine receptors (DRs) (Figure 5). Although evidence of Sig-1R modulation of these GPCRs mainly comes from in vivo and in vitro studies of the brain, they are not exclusive to the CNS since their expression and activation in the heart and kidneys mediate essential physiological and pathological mechanisms in these organs [150,151,152,153].

Sig-1R is a non-opioid receptor, and its ligands do not compete for opioid receptor binding. However, Sig-1R stimulation with agonists diminishes opioid analgesia, and antagonists binding to Sig-1R or Sig-1R silencing using siRNAs enhance opioid responses [154]. Similar responses of Sig-1R have been observed for mAchRs [7]. Moreover, Sig-1R physically associates with MORs, implying a direct interaction between the proteins. Finally, it is worth noting that Sig-1R also modulates MOR-induced analgesia through interactions with the GluN1 subunit of the glutamate N-methyl-D-aspartate receptor (NMDAR), an ion channel type of receptor for glutamate [155].

Sig-1R ligand binding augments NMDAR performance through direct interaction with its GluN1 subunits, though stimulation with Sig-1R agonists also increases the interaction of Sig-1R and GluN2 subunits [156,157,158,159]. The CBR1 is a GPCR for endocannabinoids that regulates NMDAR activity. In wild-type mice, the CBR1-NMDAR regulatory association remains unaffected by Sig-1R ligands [160]. However, in experimental NMDAR hypofunction, Sig-1R antagonists release NMDAR from the negative control exerted by CBR1 to avoid glutamate hypofunction.

There is evidence that Sig-1R forms functional interactions with DRs 1 (D1R) and 2 (D2R) but not DRs 3 and 4. D1R plays a significant role in the behavioral effects of cocaine, even though this drug blocks the dopamine transporter [161]. Cocaine potentiates D1R-mediated adenylyl cyclase and MAPK activation, and experiments in Sig-1R transfected cells and striatal slices of Sig-1R KO mice showed that D1R-mediated ERK1/2 phosphorylation depends on Sig-1R [162]. Overactivation of this pathway leads to cell death and relies on disrupting a complex formed by Sig-1R binding to D1R and Histamine 3 heteromers upon cocaine stimulation [163]. In contrast, cocaine binding to Sig-1R-D2R inhibits downstream signaling in cells and mouse striatum, thus, suggesting Sig-1R destabilizes the balance of D1 and D2 inputs by increasing D1R-mediated cAMP production and the pro-reward pathway while limiting D2R signaling [164]. Additionally, Sig-1R and D1R downstream of DOR mediate the anxiolytic effect of rubiscolin-6, a delta-opioid peptide with analgesic effects [165].

#### 5.3.4. Cell-Matrix Interactions

Integrins are transmembrane linkers used by cells to connect their actin cytoskeleton to the extracellular matrix [166]. Sig-1R associates with β1 integrin in cholesterol-enriched lipid rafts to modulate cell adhesion (Figure 5). Lipid rafts are essential for this interaction since cholesterol depletion decreases β1 integrin density and cell adhesion. Furthermore, Sig-1R stimulation with its agonist SKF-10047 or Sig-1R silencing produces similar effects that reduce cell adhesion to matrix components [57].

## 6. Sig-1R Signaling Regulates Organ Function and Disease

### 6.1. Heart

Sig-1R is vital for cardiovascular homeostasis, and its expression in cardiomyocytes and parasympathetic intracardiac neurons is well documented [167,168,169,170,171]. Immobilization and hypoxia are potent stressors that increase Sig-1R cardiac expression. In addition, IP3Rs are known modulators of Sig-1R in the heart since silencing type 1 and 2 IP3Rs lead to decreased Sig-1R mRNA levels [172].

Characterization of Sig-1R KO mice determined that the absence of these receptors promotes the development of cardiac contractile dysfunction and structural deficit during aging [12]. Furthermore, hearts from homozygous (Sig-1R-/-) mice develop cardiac fibrosis, accumulate irregularly shaped mitochondria, and have defects in mitochondrial respiratory function compared with hearts from wild-type mice. The cardiac phenotype of Sig-1R KO mice highlights its potential to regulate cardiac cellular respiration and mitochondrial organization through alterations in mitochondrial fission and fusion. The dynamin-related protein 1 (DRP1) and the optic atrophy 1 (OPA1) mitochondrial dynamin-like GTPase are protein controllers of mitochondrial fission and fusion. DRP1 phosphorylation on Ser637 inhibits its translocation from the cytosol into the mitochondrial outer membrane and is significantly increased in Sig-1R-/- cardiac cells, correlating with impaired mitochondrial fission. Similarly, higher levels of OPA1 coincide with the heightened mitochondrial fusion observed in the Sig-1R KO cells.

In the heart, Sig-1R ligands modulate contractility by altering intracellular calcium concentrations in the cardiomyocytes in a dose- and time-dependent manner [173]. Exposing cardiomyocytes to haloperidol causes a concentration-dependent increase in the amplitude of cellular contraction [174]. Additionally, age appears to contribute to the intricate effects of Sig-1R ligands in preclinical models. Nanomolar concentrations of Sig-1R ligands increase contractility and intracellular calcium levels from opening membrane calcium channels in isolated myocytes from newborn rats and ER calcium stores in myocytes from adult animals [170,174].

In intracardiac neurons, Sig-1R activation reversibly blocks delayed outwardly rectifying potassium channels and large conductance calcium-sensitive potassium channels to depress their excitability and block parasympathetic input to the heart [167]. Moreover, Sig-1R ligands exhibit pro- and anti-arrhythmic effects attributed to differential inhibition of voltage-gated Nav1.5 sodium channels and potassium channels that exert reverse responses on cardiac rhythm [131,167]. Stracina et al. studied the effects of long-term haloperidol administration on cardiac function and found a significant decrease in the relative heart rate and the prolongation of QT interval of the isolated hearts from the haloperidol-treated animals accompanied by increased expression of Sig-1R and IP3Rs in cardiac atria [175]. Altered calcium signaling in cardiomyocytes might be responsible for the enhanced sensitivity of cardiac cells to arrhythmias after haloperidol treatment [175,176].

**Figure 6:**
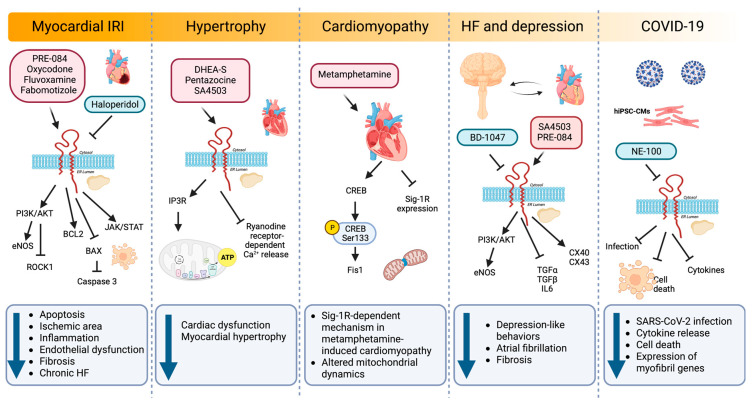
Sig-1R signaling pathways regulating cardiac injury. Sig-1R agonists and antagonists in experimental and preclinical models of myocardial IRI, hypertrophy, cardiomyopathy, depression, and COVID-19 activate diverse signaling pathways that impact cellular processes such as apoptosis, inflammation, endothelial dysfunction, fibrosis, hypertrophy, mitochondrial dynamics, atrial fibrillation, and gene expression.

In addition to Sig-1R roles in heart physiology, growing evidence points to the cardioprotective effects of Sig-1R signaling modulation in preclinical models of cardiac injury, including myocardial IRI, HF, hypertrophy, cardiac fibrosis, cardiomyopathy, depression, ER stress, and cardiovascular complications associated with SARS-CoV-2 infection (Figure 6) [177].

#### 6.1.1. Myocardial IRI and HF

Myocardial infarction is the prevailing cause of HF and often leads to cardiomyocyte apoptosis, interstitial fibrosis, and hypertrophy [178]. IRI results from the conflicting exacerbation of cellular dysfunction and death from blood flow restoration to ischemic tissues after myocardial infarction. In a model of myocardial IRI, PRE-084 maintained cardiac function by ameliorating myocardial apoptosis through BCL2 upregulation combined with BAX and caspase-3 downregulation [16]. In addition, PRE-084 treatment significantly increases the phosphorylation of AKT and endothelial nitric oxide synthase (eNOS), indicating that Sig-1R activation exerts its cardioprotective role through the AKT/eNOS pathway. Similarly, another Sig-1R ligand, oxycodone, protects cardiomyocytes and endothelial cells from IRI-induced apoptosis via activation of the PI3K/AKT pathway and the inhibition of the Ras homolog family member A (RHOA)/Rho Associated Coiled-Coil Containing Protein Kinase 1 (ROCK1) signaling [17,18,20]. Furthermore, oxycodone increased Sig-1R expression and improved myocardial function by reducing the ischemic area, the inflammatory response, and the endothelial dysfunction in a rat myocardial IRI model [18].

In a post-infarction HF model, chronic Sig-1R stimulation with fluvoxamine recovers cardiac dysfunction and ventricular remodeling [19]. Sig-1R promotes angiogenesis by activating the Janus kinase 2 (JAK2)/signal transducer and activator of the transcription 3 (STAT3) signaling pathway, further alleviating interstitial fibrosis and cardiomyocyte apoptosis. Similarly, fabomotizole hydrochloride, a Sig-1R agonist with marked anti-arrhythmic and anti-fibrillation activity, reduces the ischemic area and prevents the development of chronic HF after myocardial infarction caused by left coronary artery ligation [21]. Fabomotizole hydrochloride also decreases markers of chronic HF, including plasma levels of brain natriuretic peptide (BNP), angiotensin receptor subtype 1a (AT1AR), vasopressin receptor 1A (V1AR), glucocorticoid receptors, and exchange protein activated by cyclic-AMP 2 (EPAC2).

#### 6.1.2. Hypertrophy

Cardiac hypertrophy is a primary characteristic of several CVDs in which the heart remodels, and the cardiomyocytes increase in size in response to mechanical or pathological stresses. The pathological progression of cardiac hypertrophy initiates by ligand-stimulated membrane-bound receptors and biomechanical stress sensors and depends on signaling pathways such as calcium/calmodulin-dependent kinase II (CaMKII) signaling, RAS/RAF/MEK/MAPK cascade, and ER stress [142,179,180,181,182].

In animal models of pressure overload hypertrophy induced by transverse aortic constriction (TAC), Sig-1R activity decreases in the left ventricle, which correlates with cardiac dysfunction [22,23,24,169,183]. Treatment with DHEA-S and pentazocine confer cardioprotection in these models of pressure-overload-induced cardiac dysfunction [24,169]. Sig-1R stimulation with pentazocine ameliorates cardiac dysfunction by restoring IP3R-mediated mitochondrial ATP production and suppressing ryanodine-induced calcium release from the sarcoplasmic reticulum [23]. Likewise, synthetic Sig-1R agonists fluvoxamine and SA4503 improve cardiac function and reduce myocardial hypertrophy after TAC [24,169,177,184].

Instead of using agonist and antagonist ligands to elucidate Sig-1R function in cardiomyocyte hypertrophy, Bao Q et al. looked at microRNAs (miRNAs) regulating Sig-1R expression [183]. The induction of cardiac hypertrophy by TAC in vivo or angiotensin II (AngII) in neonatal rat cardiomyocytes reduces Sig-1R expression. In this setting, the microRNA miR-297 downregulates Sig-1R expression by directly targeting its 3′ untranslated region (3′ UTR) and promotes cardiac hypertrophy partially through XBP1 splicing and ATF4 activation.

#### 6.1.3. Cardiac Fibrosis

Fibrosis is a pathophysiological partner of CVDs as chronic injury continually activates myofibroblasts and promotes extracellular matrix buildup to develop scar tissue [185]. A fluorescence-microscopy-based high-throughput screening identified haloperidol as a potent inhibitor of myofibroblast activation, an effect dependent on Sig-1R but not transforming growth factor beta (TGF-β) [186]. In vitro, haloperidol exerts its actions by regulating intracellular calcium and inducing the ER stress response through phosphorylation of PERK and eIF2-α. Additionally, haloperidol upregulates the UPR genes ATF4, FKBP Prolyl Isomerase 11 (FKBP11), growth arrest and DNA damage-inducible protein (GADD34), and CHOP, while downregulating the neurogenic locus notch homolog protein 1 (NOTCH1) signaling genes hes family bHLH transcription factor 1 (HES1), hes related family bHLH transcription factor with YRPW motif 1 (HEY1), cyclin D1 (CCND1), and NOTCH1.

Moreover, haloperidol also ameliorates fibrosis in a mouse model of myocardial infarction in which treatment with this Sig-1R antagonist for ten days post-infarction results in a significant size reduction of the fibrotic scar and the number of myofibroblasts [186].

#### 6.1.4. Cardiomyopathy

Methamphetamine, an addictive psychostimulant drug, alters dopamine neurotransmission, and its abuse is associated with CVDs and the development of cardiomyopathy [187]. Sig-1R is a direct methamphetamine target, and its activation with PRE-084 lessens methamphetamine-induced psychomotor responses and drug-seeking behavior by limiting dopamine efflux in dopaminergic neurons [188,189]. To understand the molecular aspects of adverse cardiac remodeling in the hearts of methamphetamine users, Abdullah CS et al. used a mouse model of methamphetamine-induced cardiomyopathy and found that methamphetamine administration for several days, followed by a period of abstinence, mimics the fibrotic remodeling observed in humans and reactivates the fetal gene program characteristic of hypertrophy by upregulating cardiac atrial natriuretic peptide (ANP), BNP, and myosin heavy chain beta (MHC-β) expression [25]. Moreover, methamphetamine decreases Sig-1R levels in cardiomyocytes and alters mitochondrial dynamics by reducing the cAMP-response-element-binding protein (CREB)-mitochondrial fission 1 protein (FIS1) axis in a Sig-1R dependent manner. CREB is phosphorylated on Ser133 by protein kinase A (PKA) to allow the recruitment of CREB-binding protein and p300 and increase the transcription of CREB-dependent genes [190]. CREB binds to the FIS1 gene promoter region in cardiomyocytes to induce its transcription. Hence, alteration of CREB/pCREBSer133/FIS1 signaling in cardiomyocytes affects mitochondrial dynamics, morphometry, and respiration, providing evidence for a Sig-1R-dependent pathogenic mechanism in methamphetamine-induced cardiomyopathy.

#### 6.1.5. Relationship between Sig-1R, HF and Depression

Sig-1R agonist ligands amplify protective mechanisms by promoting the activity of trophic factors that directly or indirectly modulate pathological processes [191]. Depressive symptoms are a small but independent risk factor for CVDs, and patients with major depressive disorder (MDD) are susceptible to developing arrhythmias [192]. Sig-1R not only participates in MDD pathophysiology but also has antiarrhythmic properties, as evidenced by models of MDD induced in rodents by chronic unpredictable mild stress (CUMS) [26]. MDD rodents exhibit lower transient outward potassium current (Ito) and L-type calcium current (ICa-L) amplitudes, depression, and prolonged corrected QT (QTc) interval. Sig-1R stimulation with fluvoxamine alters the current kinetics of Ito and hyperpolarizes the steady-state activation of ICa-L while improving depression and prolonging the QTc interval under CUMS [26]. The Sig-1R also decelerates Ito inactivation and accelerates Ito recovery by activating calcium/CaMKII, effects blocked by the Sig-1R antagonist BD-1047 [27]. Furthermore, BD-1047 significantly shortens the atrial effective refractory period, and action potential duration augments atrial fibrillation inducibility and sympathetic activity and reduces parasympathetic activity and heart rate variability. A dramatic reduction in Sig-1R, connexin 40 (CX40), and Cav1.2 expression and AKT/eNOS phosphorylation accompanies these effects [193].

Additionally, sympathetic activity increases in rodents with depression-like behaviors while parasympathetic activity, heart rate variability, and atrial and hippocampal Sig1-R expression decrease [194]. Furthermore, intragastric administration of SA4503 in MDD rats improves depression-like behaviors and electrical remodeling, lowering atrial fibrillation and fibrosis frequency. Similarly, in a chronic mild stress model of atrial arrhythmias induced by burst stimulation, the Sig-1R agonist SA4503 partially reduces the incidence and duration of atrial arrhythmias in the depression group by improving the conduction function, upregulating the gap junction proteins CX40 and connexin 43 (CX43), and reducing the levels of the inflammatory mediators transforming growth factor alpha (TGF-α), TGF-β, and interleukin 6 (IL6) [195].

After myocardial infarction, mice exhibit depression-like behavior, decreased cardiac function, and lower Sig-1R expression in the hypothalamus and hippocampus. Intracerebroventricular infusion of PRE-084 in these mice rescues Sig-1R expression, reduces sympathetic activity, and improves cardiac function [28]. Meanwhile, mice treated with aortic banding and fed a high-salt diet to accelerate cardiac dysfunction also display higher sympathetic activity, increased left ventricular dimensions, and significantly lower Sig-1R expression in the brain [196]. PRE-084 also increases brain Sig-1R expression, lowers sympathetic activity, and improves cardiac function in this model. In contrast, BD-1063, a Sig-1R antagonist, increases sympathetic activity and depression-like behavior while decreasing cardiac function without cardiac injury. These studies highlight the link between Sig-1R and HF via sympathoexcitation and mental disorders.

#### 6.1.6. ER Stress

In heart disease, UPR activation to restore ER homeostasis in response to misfolded protein buildup is characteristic [197]. Alam et al. demonstrated that Sig-1R is a component of the adaptive IRE1-XBP1 ER-stress response and exerts cytoprotective effects in neonatal rat ventricular cardiomyocytes by inhibiting CHOP expression [198]. Sig-1R knockdown significantly increases CHOP expression and toxicity in cells with sustained activation of ER stress induced with tunicamycin. In contrast, Sig-1R overexpression is cytoprotective, and this effect relies on higher IRE1 phosphorylation, XBP1 expression, and nuclear transport.

#### 6.1.7. SARS-CoV-2

COVID-19, an infectious disease caused by SARS-CoV-2, can induce cardiac arrest, myocarditis, arrhythmias, cardiomyopathy, cardiogenic shock, and HF [199]. In addition, systemic inflammation, hypoxia, interferon-mediated immune responses, and plaque destabilization promote cardiac dysfunction once SARS-CoV-2 infects cardiomyocytes. Sig-1R affects the early steps of viral replication, and its ligands are antiviral compounds against SARS-CoV-2 identified by in vitro drug repurposing screens [200,201].

Salerno et al. studied the impact of Sig-1R inhibition during SARS-CoV-2 infection in human induced pluripotent stem cell-derived cardiomyocytes (hiPSC-CMs) [29]. Sig-1R localizes in the nucleus and the perinuclear region of hiPSC-CMs in proximity with voltage-dependent anion channel 1/porin, a mitochondrial marker, and calnexin, an ER chaperone. Pretreatment of hiPSC-CM with the Sig-1R antagonist NE-100 decreases SARS-CoV-2 infection while limiting cytokine release and cell death through mechanisms independent of angiotensin-converting enzyme 2 (ACE2) availability. Sig-1R antagonism also downregulates the expression of myofibril-associated genes, alters cytoskeletal integrity, and reduces cardiomyocyte beating frequency.

### 6.2. Kidney

**Figure 7:**
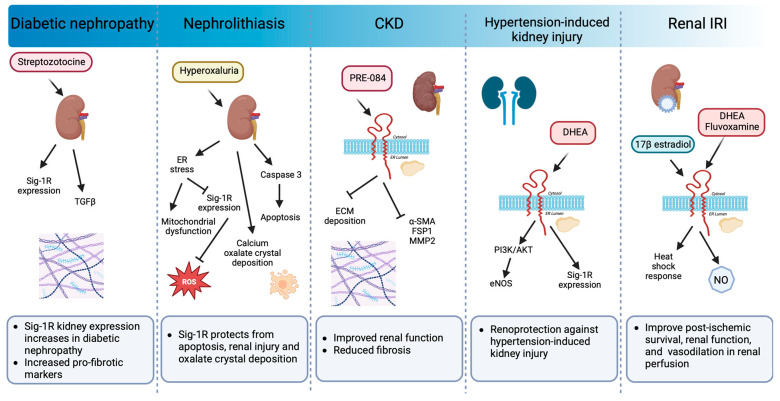
Sig-1R signaling in preclinical models of renal pathology. Sig-1R expression increases in diabetic nephropathy. Sig-1R protects from apoptosis, renal injury, and oxalate crystal deposition in a model of nephrolithiasis. Similarly, the administration of Sig-1R agonists improves renal function and reduces kidney injury and fibrosis in preclinical models of CKD, hypertension-induced kidney injury, and IR.

The kidney expresses Sig-1R more abundantly in the renal cortex compared to the medulla and the papilla, particularly in the proximal tubules but not in the glomeruli [31,202]. In addition, changes in Sig-1R renal expression and activation during postnatal maturation and in the pathogenesis of various nephropathies demonstrate its relevance [203]. Moreover, there is experimental evidence linking Sig-1R with kidney injury and sex-dependent renoprotection (Figure 7).

In a preclinical model of streptozotocin-induced diabetic nephropathy, a significant increase in Sig-1R was detected two months after induction in distal kidney tubules in conjunction with higher levels of TGF-β, an essential mediator of renal fibrosis [203]. In contrast, ER stress-linked mitochondrial dysfunction reduces MAM-associated Sig-1R levels triggering apoptosis, renal injury, and calcium oxalate crystal deposition in a model of hyperoxaluria-induced nephrolithiasis [204]. Furthermore, treatment with 4-phenylbutyrate, an ER stress inhibitor, restores Sig-1R protein expression while improving renal function and reducing oxidative markers. In addition, Haritha CV et al. administered the Sig-1R agonist PRE-084 to adenine-fed rats and demonstrated that Sig1-R activation partly reduces renal dysfunction and extracellular matrix deposition [32]. However, their study did not evaluate Sig-1R expression in the kidneys in response to adenine-induced damage.

The incidence of CKD in women increases after menopause, and significant evidence supports the protective effect of ovarian sex hormones against disease development and progression. In postmenopausal women, low DHEA levels associate with renal injury, endothelial dysfunction, and higher cardiovascular mortality. Furthermore, DHEA protects against hypertension-induced kidney injury through Sig-1R up-regulation and AKT/eNOS signaling activation in ovariectomized rats [205]. Similarly, in renal IRI, the leading cause of delayed graft function or graft loss after transplantation, the female hormonal environment positively influences the ability to recover from ischemic injury compared to males and ovariectomized females, and a post-ischemic increase in Sig-1R levels accompanies this protection [33,206]. 17β-estradiol activates Sig-1R and provides renoprotection by enhancing the heat shock response [33]. Meanwhile, Sig-1R agonist ligands such as DHEA and fluvoxamine improve post-ischemic survival, renal function, and structural damage via Sig-1R activation in proximal tubular cells, directly affecting nitric oxide (NO) production and leading to favorable vasodilation in renal perfusion [31].

## 7. Expectations of the Therapeutic Use of Sig-1R Agonists and Antagonists

Given the ineffective response or the side effects of some proven disease-modifying treatments, there is a need to find new therapeutic targets for interventions in patients affected by cardiovascular and renal diseases. Preclinical models evaluating the effects of agonist and antagonist ligands that modulate Sig-1R activity have allowed us to widely understand their contribution to cytoprotective, reparative, and anti-inflammatory mechanisms, as well as the promise that their use projects for interventions in cardiovascular and renal diseases [16,17,18,19,20,21,22,23,24,25,26,27,28,29,30,31,32,33].

Pridopidine, ANAVEX2-73, SA4503, S1RA, and T-817MA (Table 1) are five drugs with Sig-1R ligand properties currently in clinical trials and showing significant results for pain, neurodegenerative diseases, depression, and ischemic stroke [207]. Moreover, the use of SA4503, a Sig-1R agonist, and Sertraline, a selective serotonin reuptake inhibitor (SSRI) and Sig-1R antagonist, in clinical trials for ischemic stroke and depression in patients with HF illustrate the clinical potential of Sig-1R modulation in CVD [208,209,210,211,212,213,214,215,216].

Fluvoxamine has significant beneficial effects associated with improved cardiac function, ventricular remodeling, and myocardial hypertrophy reduction in preclinical HF and TAC models [19,24,169,177,184]. However, fluvoxamine translation into potential treatments of CVD in humans is still lacking. Moreover, clinical studies on fluvoxamine effects on cardiac function are scant though it is an SSRI often prescribed to elderly depressed patients with a high prevalence of cardiac disorders [217]. A double-blind, randomized trial systematically assessed cardiac function by echocardiography in middle-aged and elderly depressed patients treated with SSRIs. Fluvoxamine administration in patients previously diagnosed with myocardial infarction or hypertension exhibited significant improvement in left ventricular ejection fraction [218]. Fluvoxamine and Sertraline also have anti-inflammatory activity in human endothelial cells [219]. In patients with chronic HF, SSRI administration reduced monocyte adhesion to the endothelium and the circulating levels of vascular adhesion molecules. However, Lekakis et al. only evaluated the cardioprotective effects of SSRIs in patients for Sertraline. Orlando et al. also administered fluvoxamine to elderly patients with HF to investigate the impact of age and HF on the oral disposition kinetics of fluvoxamine [220]. Nonetheless, they did not evaluate fluvoxamine’s effects on cardiac function. Fluvoxamine also improves post-ischemic survival in animal models of kidney injury, suggesting a potential use in clinical trials for patients with AKI and transplantation [31]. Unfortunately, there are no clinical trials with SSRIs evaluating renal outcomes, and more preclinical research is needed to take Sig-1R ligands from the bench to the patient.

Furthermore, Sig-1R ligands like haloperidol, an antipsychotic, have appeared in screens of COVID-19 therapy and fibrosis aimed at repurposing approved drugs or drugs in clinical development, an advantageous approach to reduce the times required for de novo drug discovery.

Targeting Sig-1R in COVID-19 patients, though not expected to reduce established viral replication, might hinder the early steps of virus-induced host cell re-programming, decelerate the course of infection, and prevent disease exacerbation by allowing the maturation of protective immune responses [200]. A multicenter observational study found that daily haloperidol administration for over a week may not be associated with the risk of intubation or death in adult patients hospitalized for COVID-19. However, the study evaluated only one dose and did not look at systemic inflammation or cardiovascular and renal endpoints. In contrast, a double-blind, randomized, contactless clinical trial in adult outpatients with symptomatic COVID-19 showed that patients treated with fluvoxamine have a lower likelihood of clinical deterioration over fifteen days compared to placebo [221]. The use of antidepressants has been associated with lower levels of circulating inflammatory mediators implicated in COVID-19 severity [222]. Moreover, the SSRI fluvoxamine has emerged as a potential prophylactic drug for patients with SARS-CoV-2 infection [223,224]. The administration of 50 mg of fluvoxamine twice daily in a prospective cohort of COVID-19 patients resulted in zero participants with residual symptoms at 14 days compared to 60% of participants without treatment [225]. Similarly, 100 mg of fluvoxamine twice daily for ten days among high-risk adult outpatients with early diagnosed COVID-19 reduced the demand for hospitalization in a placebo-controlled, randomized trial [226]. Additionally, in an open-label, prospective cohort trial, Calusic et al. evaluated the safety and efficacy of administering 100 mg of fluvoxamine three times daily for 15 days in addition to standard therapy to COVID-19 patients in the intensive care unit (ICU). Although fluvoxamine administration at later stages failed to improve the length of ICU stay and the time on ventilator support, it reduced overall mortality [227]. Furthermore, a reduced relative risk of mortality associated with the use of SSRIs, fluoxetine, and fluvoxamine, in a retrospective multicenter cohort study analyzing electronic health records of 83,584 diverse patients diagnosed with COVID-19 [228]. Although fluvoxamine has shown promise in preventing COVID-19 progression as an early treatment option in several clinical trials, the specific contribution of fluvoxamine’s Sig-1R agonism to the advantageous effects observed in patients with SARS-CoV-2 infection remains to be established.

Additionally, a screen in transgenic animals expressing red fluorescent protein controlled by the Smooth muscle alpha-actin (α-SMA) promoter also identified haloperidol as a drug involved in primary fibroblasts differentiation into myofibroblasts by binding to Sig-1R, supporting its repurposing for treating profibrotic diseases [186].

The UPR is strongly induced in CVDs and renal diseases, and multiple preclinical studies support the idea that appropriate pharmacological modulation could impede their start and progression [229,230]. Furthermore, the induction of XBP1 preserves myocyte viability and contractility while protecting the heart from myocardial infarction in vivo; thus, Sig-1R-dependent activation of the IRE1-XBP1s pathway also has tremendous translational potential cardiac pathological conditions associated with protein misfolding [231].

In addition to the use of agonist and antagonist ligands, there is research into Sig-1R post-transcriptional regulation. For example, the microRNA miR-297 downregulates Sig-1R during the induction of cardiomyocyte hypertrophy, creating another opportunity for the possibility of microRNA therapeutics as Food and Drug Administration-approved small RNA drugs start to enter clinical medicine and expand their applications [183,232].

## 8. Concluding Remarks

Sig-1R is a ligand-activated chaperone protein predominantly residing in the ER MAM. Its subcellular location often alters upon activation to allow its communication with other signaling proteins in intracellular organelles and the plasma membrane. Interestingly, Sig-1R modulates many signaling pathways partly due to its ability to bind lipids and remodel membrane microdomains. Sig-1R ligands can act as agonists or antagonists, inducing distinct oligomeric states in the receptor to elicit different cellular responses. Sig-1R binding of antagonists favors the higher-order receptor oligomers and the formation of a complex between Sig-1R and BiP. In contrast, Sig-1R agonists promote monomeric and dimeric states to facilitate BiP dissociation, which is decisive for Sig-1R activity, interaction with other proteins, and activation of downstream signaling pathways.

In the ER MAMs, Sig-1R modulates calcium signaling between the ER and the mitochondria via IP3Rs, plays a dual role in regulating oxidative stress responses, and acts as an ER lipid scaffolding protein able to interact with and remodel cholesterol-rich microdomains. Additionally, Sig-1R participates in mitochondrial bioenergetics, mitochondrial metabolism, oxidative stress, ER stress, and autophagy, all closely linked to cell survival and apoptosis. Meanwhile, Sig-1R localization in the nuclear envelope and inside the nucleus regulates transcription and nuclear transport. At the plasma membrane, Sig-1R interacts with ion channels, RTKs, GPCRs, and integrins to control their activities and elicit intracellular signaling cascades. Evidence into Sig-1R function and signaling pathways mostly comes from the CNS, not cardiac and renal cells and tissues. However, since much of Sig-1R signaling depends on cell type, more experimental and preclinical studies on these organs are needed to understand better the receptor’s role in modulating all these cascades in health and disease.

Cardiomyocytes and parasympathetic intracardiac neurons express Sig-1R, and this receptor is essential for cardiovascular homeostasis. Sig-1R ligands modulate cardiomyocyte contractility and have pro- and anti-arrhythmic effects. Moreover, the cardiac phenotype of Sig-1R KO mice emphasizes Sig-1R’s potential to regulate cardiac cellular respiration and mitochondrial organization through alterations in mitochondrial fission and fusion. Notably, the cardioprotective effects of Sig-1R modulation are evident in preclinical models of myocardial IRI, HF, hypertrophy, cardiac fibrosis, cardiomyopathy, depression, ER stress, and COVID-19. The kidney also expresses Sig-1R, and changes in its expression during the pathogenesis of various nephropathies demonstrate its relevance as a molecular target. Moreover, experimental evidence links Sig-1R with kidney injury and sex-dependent renoprotection.

Although extensive, the evidence from preclinical models of cardiac and renal injury has yet to unfold the complete mechanism by which the Sig-1R exerts its actions. However, the data jointly suggests that the overexpression and activation of Sig-1R are protective in the presence of damage caused by stress due to aging or pathologies. Hence, targeting Sig-1R signaling in the heart and the kidneys could be a promising alternative to prolong cardiovascular and renal disease progression that needs our attention and would benefit from increased translational research. Sig-1R ligands in preclinical models have significantly beneficial effects associated with improved cardiac function, ventricular remodeling, hypertrophy reduction, and, in the kidney, reduced ischemic damage and renoprotection. These basic discoveries could inform clinical trials for HF, myocardial hypertrophy, AKI, and CKD.

## Figures and Tables

**Table 1:** Types of Sig-1R ligands and their classification.

Ligand Type	IUPAC Name	Name	Classification	CAS
Natural Agonist	[(3S,8S,9S,10R,13S,14S,17S)-17-acetyl-10,13-dimethyl-2,3,4,7,8,9,11,12,14,15,16,17-dodecahydro-1H-cyclopenta[a]phenanthren-3-yl] hydrogen sulfate	PREG-S	Steroid sulfate	1247-64-9
	(3S,8R,9S,10R,13S,14S)-3-hydroxy-10,13-dimethyl-1,2,3,4,7,8,9,11,12,14,15,16-dodecahydrocyclopenta[a]phenanthren-17-one	DHEA	Androstanoid	53-43-0
	(8S,9S,10R,13S,14S,17S)-17-acetyl-10,13-dimethyl-1,2,6,7,8,9,11,12,14,15,16,17-dodecahydrocyclopenta[a]phenanthren-3-one	Progesterone	C21-steroid hormone	57-83-0
Synthetic Agonist	1-[2-(3,4-dimethoxyphenyl)ethyl]-4-(3-phenylpropyl)piperazine;dihydrochloride	SA4503 *	Piperazines derivative	165377-44-6
	2-morpholin-4-ylethyl 1-phenylcyclohexane-1-carboxylate	PRE-084	Phencyclidine derivative	138847-85-5
	1,2-bis(2-methylphenyl)guanidine	DTG	Guanidine derivative	97-39-2
	(1R,9R,13R)-1,13-dimethyl-10-(3-methylbut-2-enyl)-10-azatricyclo [7.3.1.02,7]trideca-2(7),3,5-trien-4-ol	Pentazocine *	Synthetic opioid	359-83-1
	(1R,9R,13R)-1,13-dimethyl-10-prop-2-enyl-10-azatricyclo [7.3.1.02,7]trideca-2(7),3,5-trien-4-ol	Alazocine, SKF-10047	Synthetic opioid	14198-28-8
	4-(3-methylsulfonylphenyl)-1-propylpiperidine	Pridopidine *	Phenylpiperidine	346688-38-8
	1-(2,2-diphenyloxolan-3-yl)-N,N-dimethylmethanamine;hydrochloride	ANAVEX 2-73 *, AE-37 hydrochloride	Blarcamesine hydrochloride	195615-84-0
	1-[3-[2-(1-benzothiophen-5-yl)ethoxy]propyl]azetidin-3-ol;(Z)-but-2-enedioic acid	T-817MA *	Edonerpic maleate	519187-97-4
	methyl (1R,2R,3S,5S)-3-benzoyloxy-8-methyl-8-azabicyclo[3.2.1]octane-2-carboxylate	Cocaine	Tropane alkaloid	50-36-2
Synthetic Antagonist	4-[4-(4-chlorophenyl)-4-hydroxypiperidin-1-yl]-1-(4-fluorophenyl)butan-1-one	Haloperidol *	Phenylbutylpiperadine derivative	52-86-8
	4-Methoxy-3-(2-phenylethoxy)-N,N-dipropylbenzeneethanamine hydrochloride	NE-100	Hydrochloride	149409-57-4
	4-[2-(5-methyl-1-naphthalen-2-ylpyrazol-3-yl)oxyethyl]morpholine	S1RA *, E-52862	Hydrochloride polymorph and solvate	878141-96-9
	N-(2-(3,4-Dichlorophenyl)ethyl)-N-methyl-2-(dimethylamino)ethylamine	BD-1047	Primary amine	138356-20-4
	1-[2-(3,4-Dichlorophenyl)ethyl]-4-methylpiperazine	BD-1063	Primary amine	150208-28-9

* Sig-1R agonists and antagonists being evaluated in clinical trials. Abbreviations: IUPAC, International Union of Pure and Applied Chemistry; CAS, Chemical Abstracts Service; PREG-S, pregnenolone sulfate; DHEA, dehydroepiandrosterone; DTG, 1,3-Di-o-tolylguanidine.

## Data Availability

No datasets were generated or analyzed during the current study.
